# Experimental Study on Lap-Spliced Performance of High-Strength Stainless Steel Wire Mesh in Engineering Cementitious Composites

**DOI:** 10.3390/ma16113959

**Published:** 2023-05-25

**Authors:** Xuyan Zou, Xiyuan Zhang, Ziyuan Li, Juntao Zhu

**Affiliations:** 1 School of Civil Engineering, Zhengzhou University of Technology, Zhengzhou 450044, China; 2 School of Water Conservancy and Civil Engineering, Zhengzhou University, Zhengzhou 450001, China

**Keywords:** Engineering Cementitious Composites (ECCs), high-strength stainless steel wire mesh (HSSSWM), lap-spliced performance, pull-out test, lap strength

## Abstract

To investigate the mechanical properties of high-strength stainless steel wire mesh (HSSSWM) in Engineering Cementitious Composites (ECCs) and determine a reasonable lap length, a total of 39 specimens in 13 sets were designed and fabricated by considering the diameter of the steel strand, spacing of the transverse steel strand, and lap length. The lap-spliced performance of the specimens was tested through a pull-out test. The results revealed two failure modes in the lap connection of steel wire mesh in ECCs: pull-out failure and rupture failure. The spacing of the transverse steel strand had little effect on the ultimate pull-out force, but it restricted the slip of the longitudinal steel strand. A positive correlation was found between the spacing of the transverse steel strand and the slip amount of the longitudinal steel strand. With an increase in lap length, the slip amount and ‘lap stiffness’ to peak load increased, while the ultimate bond strength decreased. Based on the experimental analysis, a calculation formula for lap strength considering the correction coefficient *β* was established.

## 1. Introduction

Concrete buildings, as a traditional form of architecture, inevitably experience a series of cracks during their service life due to adverse external conditions such as carbonation of concrete, freeze–thaw damage, and corrosion from corrosive media. These conditions significantly reduce the bearing capacity and service life of the structure. To enhance the durability and sustainability of concrete structures, many experts and scholars have conducted extensive and in-depth research on the performance of new building materials based on various factors. One of these materials is Engineering Cementitious Composites (ECCs). By adding fiber materials to ECCs, the fiber bridging action prevents the opening and propagation of cracks, resulting in good strain hardening behavior and multiple cracking performance compared to ordinary concrete in tension [1,2,3,4]. This improved ductility, deformation ability, and energy absorption ability of the structure [5,6]. Compared to ordinary concrete materials, ECCs exhibit excellent toughness under tensile load, with tensile strain several hundred times higher than ordinary concrete [7]. However, when used alone, ECCs exhibit relatively low strength under tensile load, and it needs to be combined with other reinforcement materials to function effectively. Ordinary steel bars have low strength, and it is difficult to fully utilize the excellent performance of ECCs. Although organic fiber materials have excellent performance, their economical and practical applicability is relatively poor due to cost constraints [8,9]. On the other hand, high-strength stainless steel strand has high strength, moderate cost, and strong practicality. The most important aspect is that it has a good ability to function effectively with ECCs, which makes high-strength steel wire mesh (HSSSWM) reinforced ECCs an ideal material with high strength, high ductility, and good economic practicality [10]. Therefore, HSSSWM-reinforced ECCs have a very broad application prospect in future engineering structural construction materials.

In recent years, as research on the internal properties of concrete has gradually deepened, numerous experts and scholars have studied the bond-slip behavior of new composite materials, yielding significant results. Lapping is the most commonly used and simplest form of material connection, but it reduces the grip of the matrix on the reinforced material, resulting in significantly lower lap strength than bond strength [11,12]. Therefore, to prevent structural failure caused by lap splicing, further research on the lap-spliced performance of materials is necessary. Li et al. [12] studied the bond stress–slip relationship between HSSSWM and ECCs, and proposed a local bond stress–slip model for HSSSWM-reinforced ECCs. Xu et al. [13] artificially rusted the reinforcement in the specimens using the constant current method, and investigated the effect of corrosion on the lap-spliced performance of the reinforcement through uniaxial tension tests. Al-Quraishi et al. [2] investigated the tension lap-spliced length of reinforcing bars embedded in reactive powder concrete (RPC). The results of the study showed that the bond strength of the spliced bars increased with the increase in lap length up to a certain point, beyond which it decreased. The authors also recommended an optimal lap length for the spliced bars embedded in RPC based on their experimental findings. Chen et al. [5] conducted a pull-out test under direct tension on a total of 27 specimens and concluded that the failure mode was governed only by the lap length of the Carbon-Fiber Reinforced Polymer (CFRP) bar embedded in Ultra-High Performance Concrete (UHPC). Through a tensile test, Choi et al. [6] studied the bond properties of overlapping steel bars in Strain-Hardening Cementitious Composites (SHCC) reinforced with a mixture of steel fibers and carbon fibers of different compressive strength and proposed a formula for calculating the average bond stress considering the impact of various factors, such as the lap length. Ma et al. [14] found that the steel bar rupture failure occurred under a rather small embedment length and proposed an equation for calculating the minimum yield lap length and the minimum ultimate lap length. Tighiouart et al. [15] investigated the effect of the diameter of Fiber Reinforced Polymer (FRP) rebar and lap length on bond stress. The experimental results showed that the number of cracks and the crack zone decreased with the increase in the lap length. Choi et al. [6] and Zemour et al. [16] have both verified that the bond strength of the strengthened beam decreases as the lap length of GFRP bars increases. Wu et al. [17] investigated the influence of stirrup spacing on the lap-spliced performance of reinforcement. Tabatabaei et al. [18] explored the compression lap length required for GFRP bars in concrete columns. Zhao et al. [19] studied the effects of various factors on the lap-spliced performance of GFRP reinforcement in concrete, and proposed equations for calculating the bond strength and lap length of GFRP reinforcement lap in concrete. Based on the aforementioned research, Zhu et al. [20] investigated the effect of transverse steel strands on the bonding performance of HSSSWM and ECCs. Zhang et al. [21] examined the influence of different types of fibers on the bonding performance of reinforced materials. They analyzed the pull-out mechanism of steel strand fibers and hook-end fibers through pull-out tests and proposed a predictive model for the interfacial bonding performance of fiber-reinforced materials. These numerical and experimental studies have enhanced our understanding of the lap performance of these specimens [22,23,24,25,26].

As previously mentioned, several studies have been conducted on the mechanical properties, failure characteristics, and lap length of various reinforcement materials [27,28,29,30,31,32,33,34]. Many of these studies have also investigated the bonding performance between HSSSWM and ECCs [35,36,37,38,39,40]. However, there are still very few studies on the lap-spliced performance of HSSSWM in ECCs. With this in mind, this paper presents a study on the lap-spliced performance of HSSSWM in ECCs. The pull-out test was conducted on specimens with different parameters, such as the spacing of the transverse steel strand, lap length, and the diameter of the steel strand. Through analyzing the experimental results, a preliminary discussion was made on the calculation method of the lap strength of HSSSWM-reinforced ECCs.

## 2. Test Specimens and Experimental Parameters

### 2.1. Materials

**Figure 1:**
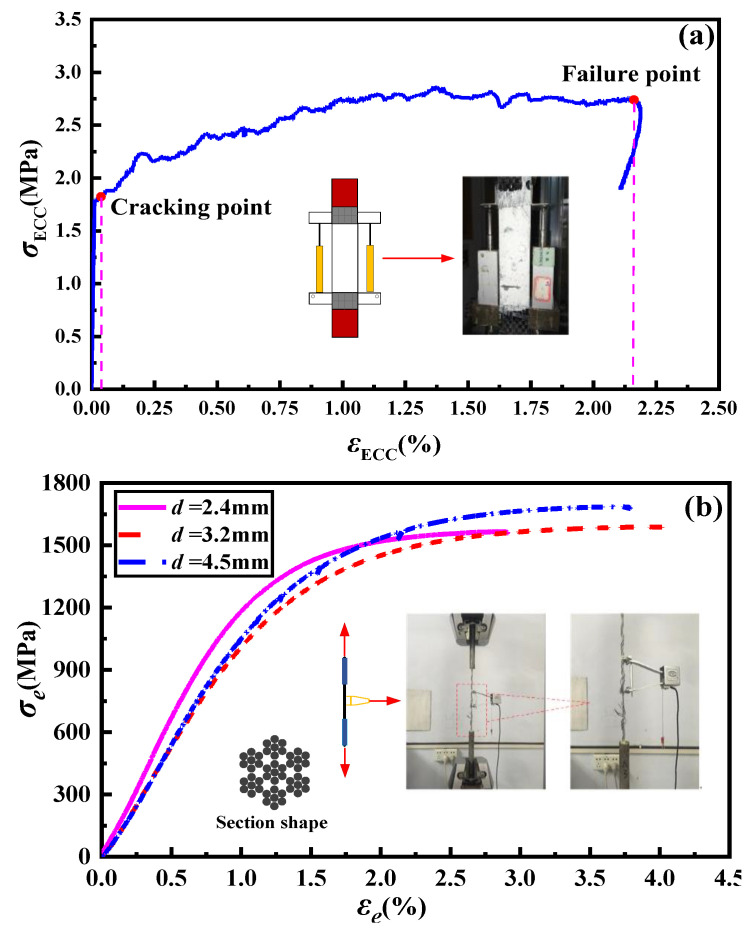
Mechanical properties of (**a**) stress–strain relationship of ECCs under tension and (**b**) stress–strain relationship of HSSSW under tension.

The composition and mix proportions of ECCs utilized in this study are listed in Table 1, and the preparation method is referenced from the literature [12]. The high-strength stainless steel strand is comprised of seven bundles of seven high-strength steel wires. Three types of steel strands, with diameters of 2.4 mm, 3.2 mm, and 4.5 mm, were chosen for testing, and their cross-sectional shape and mechanical properties are presented in Figure 1b and Table 2, respectively. Prior to the lap-spliced performance test, the material properties of the selected ECCs and steel strands were evaluated. A set of cube test blocks measuring 70.7 mm on the side was used to conduct a compression performance test on the ECCs. After 28 days of standard curing, the average compressive strength of the ECCs was found to be 32.5 MPa. For the ultimate tensile test, an ECCs plate with an averaged cracking strength and ultimate tensile strength was selected, measuring 280 mm × 40 mm × 15 mm, and its stress–strain curve is shown in Figure 1a. The average values of each group of test results were taken, with an average cracking strength of 1.8 MPa, ultimate tensile strength of 3.1 MPa, and ultimate tensile strain of 1.96%.

Uniaxial tensile tests were conducted on three types of HSSSW samples to characterize their mechanical properties. The total length of the sample was 500 mm, with an anchoring length of 150 mm at both ends and a measurement section length of 200 mm. The test was conducted using displacement control with a loading speed of 0.3 mm/min until the HSSSW was pulled off. Three identical samples of each HSSSW diameter were taken, and the average tensile results were adopted. The cross-sectional features and the measured mechanical properties of HSSSW are presented in Table 2, and the stress–strain relationship of HSSSW under tensile loading is shown in Figure 1b.

### 2.2. Specimen Design and Preparation

**Figure 2:**
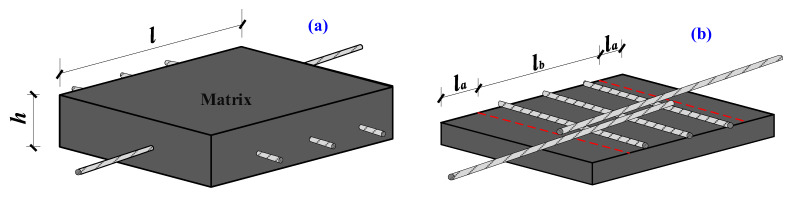
The schematics of interfacial test specimens. (**a**) Overall appearance diagram and (**b**) schematic diagram of lap-spliced HSSSW.

**Figure 3:**
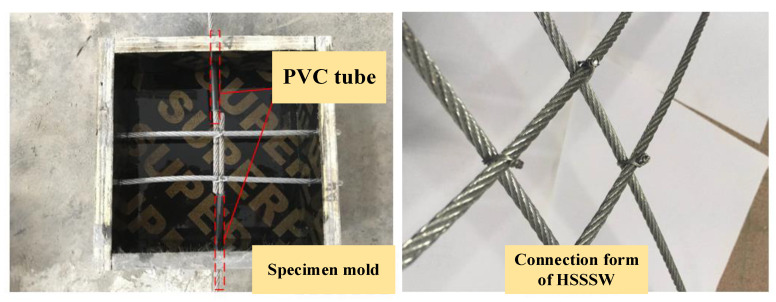
The preparation of specimens.

To guarantee the reliability of the test outcomes, three identical specimens were cast for each group, resulting in a total of 39 specimens for 13 groups of tests. A schematic diagram of the specimen is shown in Figure 2, where *l* represents the length of the specimen, *l_b_* is the lap length, *l_a_* is the length of the non-bonded section, and *h* is the thickness of the specimen. The parameter design of the specimens is presented in Table 3, taking into account the test parameters, such as the diameter of HSSSW and the lap length. The specimens were cast in wooden molds. Before pouring, all longitudinal steel strands were fixed at the height of *d*/2 above the center of the mold, while transverse steel strands were fixed at the height of *d*/2 below the center of the mold, where *d* is the diameter of the steel strand. The specimen preparation process is presented in Figure 3. The anchorage length of the specimen is determined by the length of the PVC plastic pipe. A preservative film is wrapped around both ends of the non-bonded section of the steel strands to prevent mortar from infiltrating into the non-bonded section during pouring. Before pouring the specimen, the HSSSW is tightly fixed using clamps and binding methods. After sufficient vibration during pouring, the surface is covered with a preservative film. The poured specimens are cured at room temperature for 48 h, followed by standard curing for 28 days.

### 2.3. Test Setup and Instrumentation

**Figure 4:**
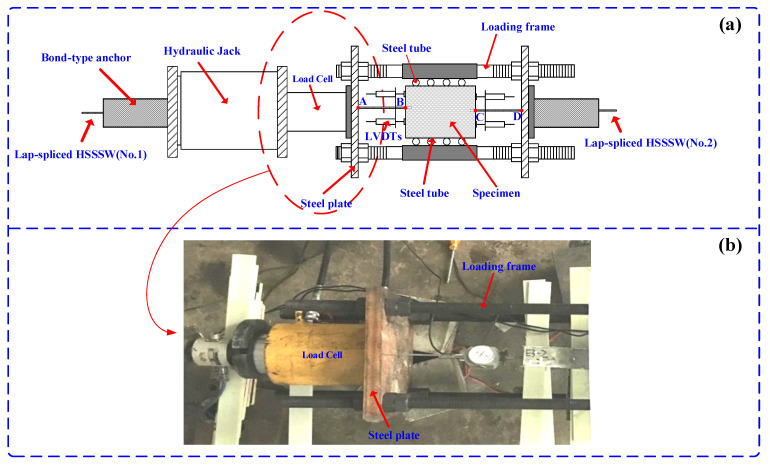
Test setup of (**a**) schematic diagram and (**b**) actual setup.

The lap-spliced specimens were loaded using a 500 kN center hole jack, as illustrated in Figure 4. The loading frame comprised two steel plates of 300 mm × 300 mm × 30 mm and four high-strength bolts with a grade of 8.8. A load cell with a capacity of 500 kN was positioned directly between the hydraulic jack and the steel plate to control the tensile load. The entire apparatus was placed horizontally, with the loading end located on the left side. The HSSSWs at both ends of the specimen were secured using a central anchoring device consisting of two steel plates with central holes. To prevent eccentric tension during testing and thereby affecting the accuracy of the results, several steel pipes were placed between the specimen and the steel plate to ensure that the specimen remained centered during the loading process. The tensile load was applied to the specimen at a constant rate of 5% maximum load until the specimen was destroyed (i.e., the steel strand was pulled off or pulled out).

As depicted in Figure 2, *l_b_* represents the actual lap length of the HSSSW in ECCs. During the loading process of the device illustrated in Figure 4a, due to the relatively significant tensile force on the AB and CD segments, the displacement of points A and D is measured using a displacement meter. Further, the displacement of points B and C is obtained through numerical calculation:

(1) $$\Delta l_{AB}=\frac{Pl_{AB}}{A_{s}E_{s}}$$

where ∆*l_AB_* is the deformation of section AB, *P* is the pulling load, *l_AB_* is the length of section AB, and *E_s_* and *A_s_* represent the elastic modulus and the actual cross-sectional area of HSSSW, respectively. The calculation method for ∆*l_CD_* is the same as described previously.

(2) $$\{\begin{array}{c} l_{1}=l_{A}-\Delta l_{AB}\\ l_{2}=l_{D}-\Delta l_{CD}\\ l_{LP}=l_{1}+l_{2} \end{array}$$

where *l*_1_ is the actual slip amount at the left end of the specimen, *l*_2_ is the actual slip amount at the right end of the specimen, and *l_A_* and *l_D_* represent the actual displacement values of point A and point D, respectively. *l_LP_* is the actual slip amount of the lap-spliced segment.

(3) $$\tau =\frac{P}{\pi dl_{b}}$$

where *τ* is the average bond stress, *d* is the diameter of the HSSSW, and *l_b_* is the lap length.

## 3. Experimental Phenomena and Results

### 3.1. Failure Mode

**Figure 5:**
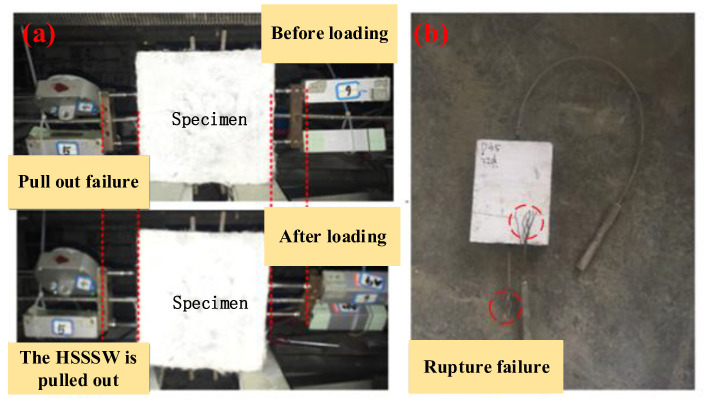
Failure mode of (**a**) pull-out failure and (**b**) rupture failure.

During the pull-out test, two main failure modes were observed: (1) the HSSSW was pulled out from the ECCs, and (2) rupture failure of the HSSSW. Figure 5 illustrates the details of these failures. The loading process of the test was controlled by force, and the linear variable displacement transducers (LVDT) on the left side of the specimen were slowly removed when the load reached approximately 90% of the peak load. However, for subsequent sliding value calculations, the sliding value of this section was determined based on the sliding trend of the previous 90% load. At the initial stage of loading, the displacement of both the loading end and the free end of the specimen slowly increases. However, as the load gradually increases, the load–slip curve of the specimen exhibits a non-linear trend. During the loading process, as the peak load of the specimen is approached, the load increases slowly while the corresponding slip value increases faster. In specimens with pull-out failure, after reaching the peak load, the HSSSW at one end of the specimen is pulled out by about 10 mm, and the load value at this point decreases to about 40% of the peak load. In specimens with rupture failure, the nonlinearity of the load–slip curve is apparent before reaching the peak load, and the HSSSW is then pulled off when the load reaches the ultimate bearing capacity of the HSSSW.

The peak loads for the three selected diameters of the HSSSW (2.4 mm, 3.2 mm, and 4.5 mm) are 4.3 kN, 7.8 kN, and 14.4 kN, respectively. When the above-mentioned failure modes occur in the lap-spliced specimens, the load decreases rapidly, and the entire process is relatively sudden, exhibiting brittle failure characteristics. After the failure, the load–slip curve decreases rapidly, and the data obtained thereafter generate relatively large errors. Therefore, only the rising section of the curve is considered in the analysis of the lap-spliced performance of the specimen.

### 3.2. Test Results and Analysis

Based on the experimental results, Table 4 presents the failure modes and key parameters that represent the lap-spliced performance for each specimen. The peak load of Group A specimens is relatively similar. However, the slip amount corresponding to the peak load for A1 to A4 are 2.99 mm, 1.57 mm, 2.03 mm, and 2.48 mm, respectively. This suggests that adding transverse steel strands to ECCs and changing their spacing has minimal impact on the peak load of the lap-spliced specimen. However, as transverse steel strands are added, and their spacing decreases, the corresponding slip amount of each group of specimens under peak load gradually decreases. This indicates that the addition of transverse steel strands to the lap-spliced specimen hinders the sliding of longitudinal steel strands, and as the spacing of transverse steel strands decreases, the restraining effect on longitudinal steel strands becomes more pronounced. In Group C, the corresponding peak loads for specimens with lap lengths of 20d, 22d, and 25d were found to be 6.35 kN, 7.02 kN, and 7.76 kN, respectively. Similar trends were observed in Groups B and D, indicating that the lap length of the steel strand has a significant impact on the lap-spliced performance. In Groups B-1, C-1, and D-1, the corresponding peak loads for specimens with HSSSW diameters of 4.5 mm, 3.2 mm, and 2.4 mm were 3.84 kN, 6.35 kN, and 11.60 kN, respectively. This indicates that the diameter of the HSSSW also has a significant impact on the lap-spliced performance of the specimen. Furthermore, some specimens exhibited steel strand rupture failure due to the tensile strength of the steel strand being less than the ultimate bonding strength. In these specimens, the lap length of the steel strand was relatively large, resulting in better lap-spliced performance. However, when the ultimate load of the steel strand was reached, the steel strand was instantly pulled apart, leading to the aforementioned failure mode.

In addition, Table 4 displays the strength conversion rate of the steel strand, which indicates the proportion of strength exerted by the steel strand at the point of specimen failure. For the specimens that experienced steel strand rupture failure, their strength conversion rates were close to 100% due to the fact that the tensile strength of the steel strand was lower than the ultimate bonding strength. Group D achieved the highest strength conversion rate among the specimens that experienced steel strand pull-out failure, demonstrating that this group of specimens could fully utilize the strength of the steel strand. Additionally, as the spacing between the transverse steel strands increased, the strength conversion rate also increased, and this trend was observed in other groups as well. The strength conversion rate presented in Table 4 could serve as a reference for designing such specimens to fully exploit the strength of the steel strand.

**Figure 6:**
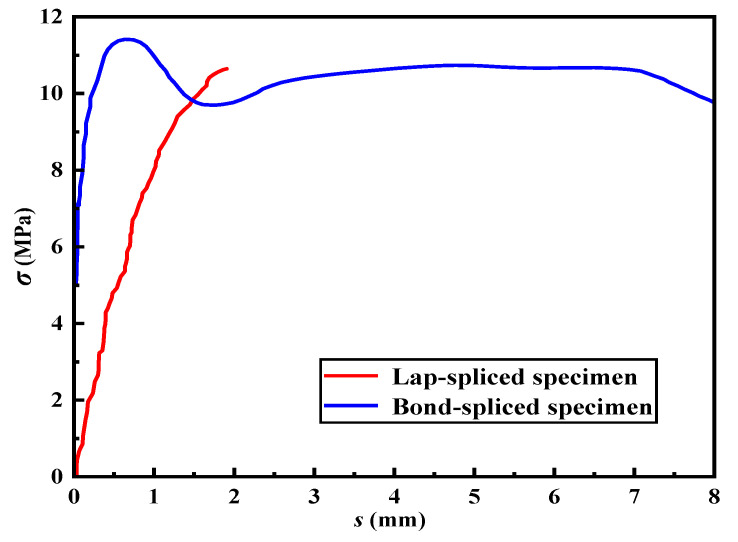
Typical bond stress–slip curves of bond-spliced specimen and lap-spliced specimen.

Referring to the existing literature on the bonding behavior testing of steel strands in the ECCs matrix [41], Figure 6 shows the typical bond stress–slip curves of the bond-spliced and lap-spliced specimens. Only the anchoring methods of the steel strands in the ECCs were changed in the two tests, while other experimental parameters were kept the same. Based on the results shown in Figure 6, it can be observed that the bond strength of the lap-spliced specimens is lower compared to that of the bond-spliced specimens. Furthermore, the relative slip of the lap-spliced specimens is higher than that of the bond-spliced specimens under the same load. The failure mode of the bond-spliced specimens is classified as ductile failure, and the process of load reduction until complete failure of the specimen is relatively slow. However, the lap-spliced specimens fail in a brittle manner, and upon failure, the slip increases rapidly, and the load decreases abruptly. There are two main reasons for the significant difference in failure mode between the two types of specimens. Firstly, due to shear action during loading, the ECCs between two longitudinally lap-spliced steel strands may be broken, which weakens the ECCs’ grip force on the steel strand and results in a lap strength significantly lower than that of the bond-spliced specimen. Moreover, the reinforcement and restraint effects of the transverse steel strand are limited, leading to a significant difference in failure mode from the bond-spliced specimen. Secondly, during the loading process, slippage occurs simultaneously from both ends of the longitudinal steel strand toward the middle. As the load increases, the amount of slippage continues to accumulate, which results in sudden pulling out of the steel strand when the lap-spliced specimen is damaged, and the process is relatively rapid. Therefore, when designing specimens using lap-spliced anchoring, it is necessary to reasonably consider design parameters, such as the lap length and transverse steel strand spacing, in order to fully utilize the strength of the steel strand and prevent premature brittle failure of the specimen under actual loading.

## 4. Influences on the Lap-Spliced Performance

### 4.1. Transverse Steel Strand and Its Spacing

**Figure 7:**
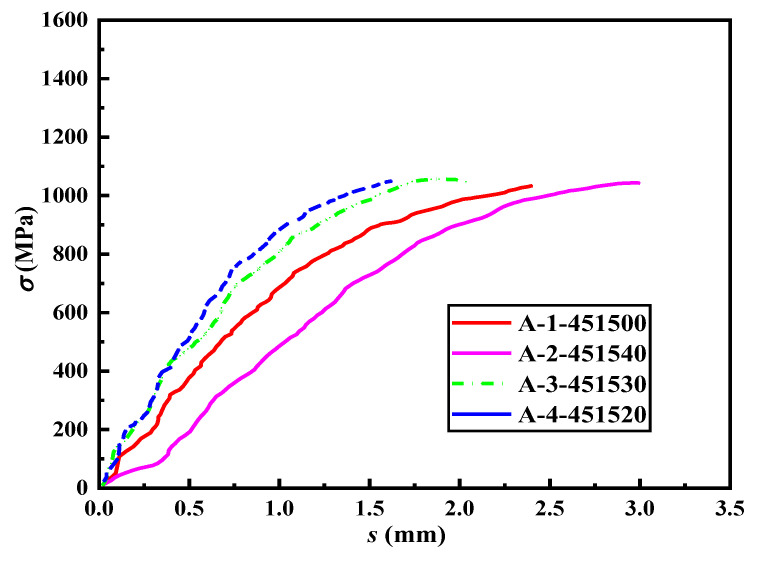
The stress–slip curves of the specimens in Group A.

**Figure 8:**
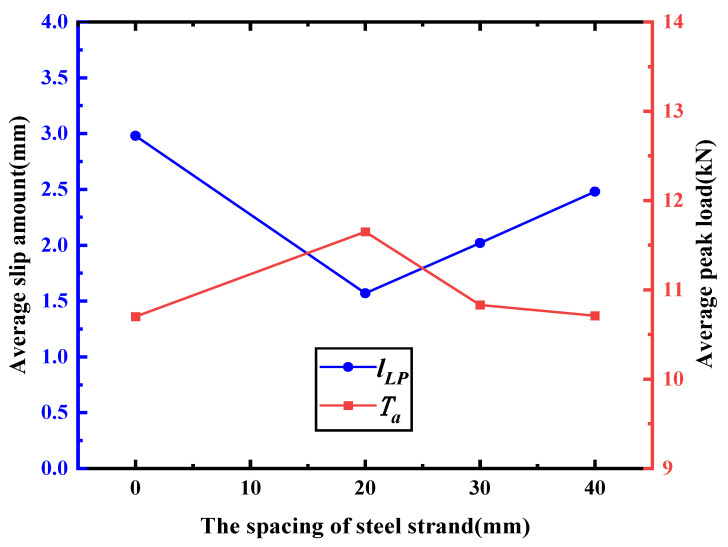
The relationship between the test results and the spacing of the steel strand.

Figure 7 displays the stress–slip curves of Group A specimens. The figure reveals that the peak load of the specimens is relatively similar, indicating that the addition of a transverse steel strand has a negligible impact on the bond strength of the lap-spliced specimens. Nevertheless, there is a significant variation in the corresponding slip amount when reaching the peak load. Moreover, under the same level of load, the corresponding slip amount of the longitudinal steel strand decreases with the reduction in the spacing of the transverse steel strand. Figure 8 illustrates the correlation between the spacing of the transverse steel strand and the average peak load *T_a_* and its corresponding average slip amount *l_LP_*. As observed from the figure, a larger spacing of the transverse steel strand leads to a greater slip amount when the peak load is reached, but it has a negligible effect on the peak load. This is mainly attributed to the fact that the addition of a transverse steel strand can restrict the sliding of the longitudinal steel strand, and a smaller spacing of the transverse steel strand leads to a better restraining effect on the longitudinal steel strand.

However, the addition of a transverse steel strand does not alter the brittle failure mode of the lap-spliced specimen. This is due to the fact that the two longitudinal steel strands in the lap-spliced specimen are positioned side by side and in close proximity. As compared to the conventional bond-spliced specimen, the surrounding ECCs have a reduced gripping effect on the steel strands, and the ECCs between the two longitudinal steel strands are subjected to two-way compression, while the transverse steel strand has a limited mitigating effect on them, resulting in brittle failure.

### 4.2. Lap Length

**Figure 9:**
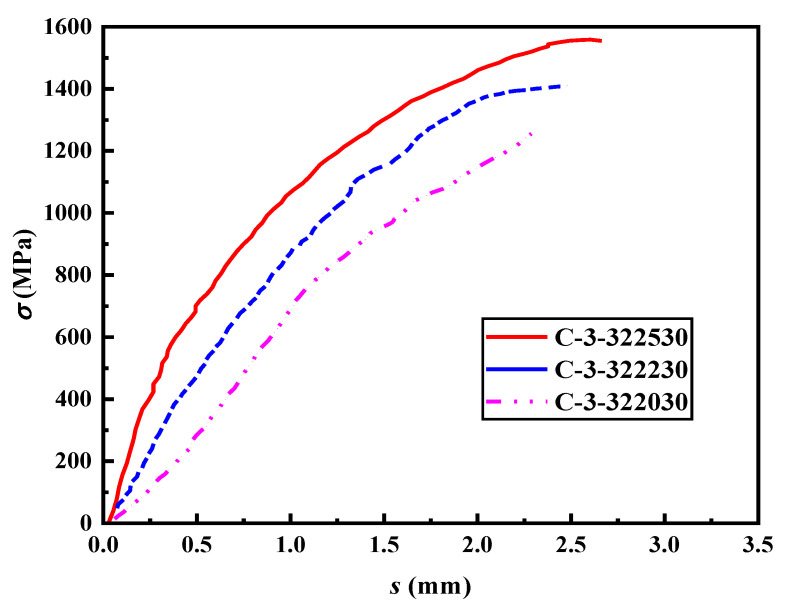
The stress–slip curves of the specimens in Group C.

**Figure 10:**
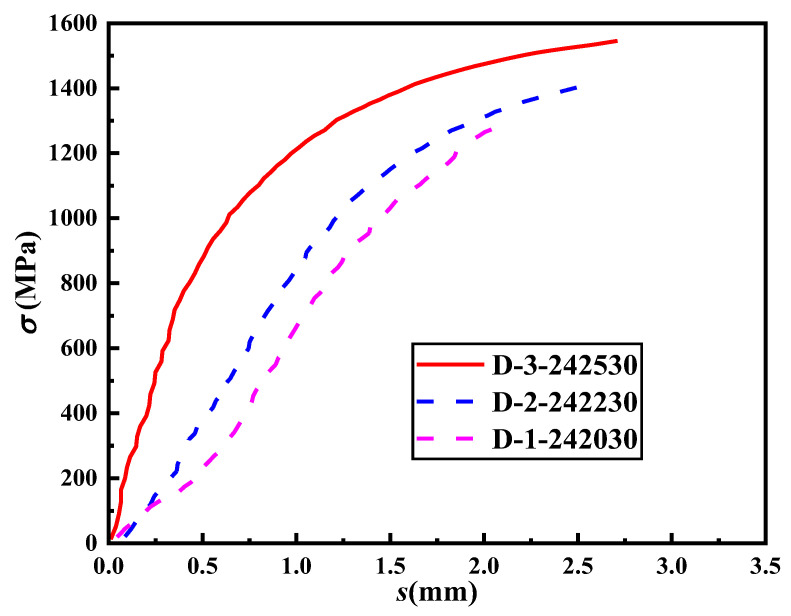
The stress–slip curves of the specimens in Group D.

The results in Table 4 suggest that the lap length has a significant influence on the lap-spliced performance of the specimens. Figure 9 and Figure 10 present the stress–slip curves for the groups of specimens with varying lap lengths. It can be observed that the slip amount corresponding to the same load decreases as the lap length increases. Moreover, during the later stage of loading, the amount of slip generated under the same load decreases with the increase in lap length, indicating that the lap strength also increases with the lap length. From the figures, it is apparent that the lap length is inversely proportional to the ultimate bond stress of the specimen. As the lap length increases, the ultimate bond stress decreases. This trend is mainly attributed to the non-uniform distribution of bond stress along the lap length during the pull-out test. With the reduction in the lap length, the relative length of the high-stress zone increases, which leads to an increase in the ultimate bond stress. Smaller lap lengths have an average bond stress closer to the actual bond strength. Additionally, based on the test results, there is a positive correlation between the relative lap length (the ratio of the lap length to the diameter of the steel strand) and the amount of slip. As the relative lap length increases, the slip amount corresponding to the peak load also increases.

### 4.3. The Diameter of Steel Strand

**Figure 11:**
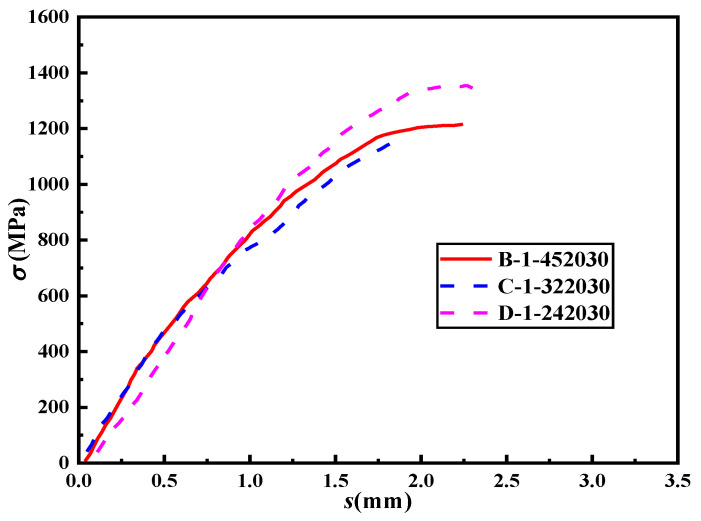
The stress–slip curves of the specimens with different diameters of steel strands.

Figure 11 presents the stress–slip curves of lap-spliced specimens with varying steel strand diameters. The figure shows that with an increase in the diameter of the steel strand, the corresponding slip under the same load decreases. Moreover, a larger diameter results in a higher ultimate tensile force, a steeper slope in the rising section of the curve, and greater lap strength. The main reason for this trend is Poisson’s effect. During the tensile process of the steel strand, the radial shrinkage deformation of the steel strand reduces the bonding performance of the specimen. As the diameter of the steel strand increases, Poisson’s effect becomes more pronounced, resulting in greater radial deformation of the steel strand and, thus, a decrease in the bond strength between the HSSSW and ECCs.

## 5. Prediction Model of Lap Strength in HSSSW-ECCs

Based on the analysis of the test results, it is evident that the lap-spliced performance of HSSSW-ECCs is dependent on various factors, such as material properties, lap length of the steel strand, the diameter of the steel strand, and transverse spacing of the steel strand. As a composite material, the lap-spliced performance of HSSSW-ECCs can be considered a comprehensive reflection of the above factors. Therefore, the lap strength can be roughly characterized as the average bond strength corresponding to the peak load. To comprehensively evaluate the impact of the aforementioned factors on the lap-spliced performance, based on the form of the interfacial bond bearing capacity calculation formula [42], the characteristic value *f_ls_* (MPa) is utilized to represent the lap strength of the HSSSW-ECCs, and the calculation model is as follows:

(4) $$\begin{array}{ccc} f_{\mathrm{ls}}=k_{f}\times \left(\frac{l}{d}\cdot \beta_{s}\cdot \sigma_{\mathrm{st}}+\sigma_{\mathrm{et}}\right)+19.38 & & \end{array}$$

where *k_f_* is the strength adjustment coefficient, *l*/*d* is the relative lap length, *l* (mm) is the lap length of the steel strand, *d* (mm) is the diameter of the steel strand, *β_s_* is the transverse steel strand influence coefficient, *σ_st_* (GPa) is the ultimate tensile strength of the steel strand, and *σ_et_* (MPa) is the ultimate tensile strength of the ECCs.

**Figure 12:**
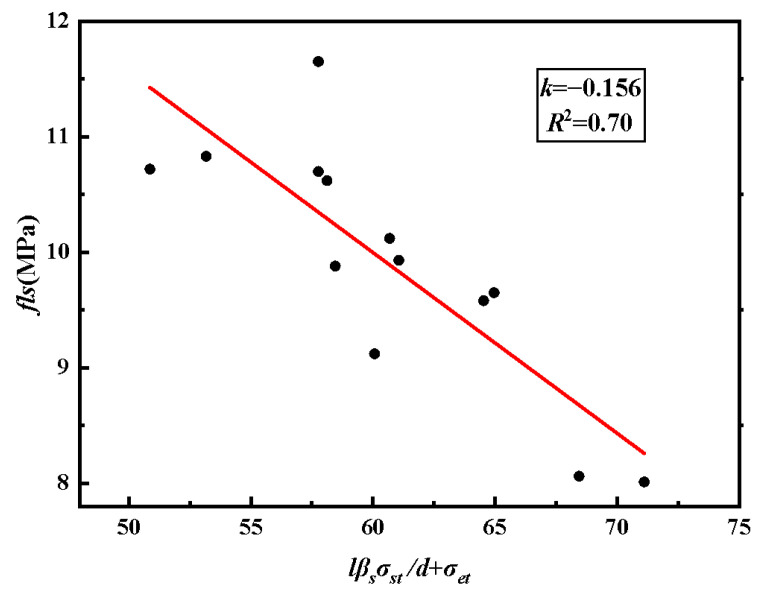
Reduction coefficient fitting.

Due to the large number of calculated variables in Equation (4), it can be processed in steps to determine the parameters in the equation. Firstly, the calculation formula for *β_s_* is shown in Equation (5). Based on the test data in Table 4, all data were used for parameter fitting. Linear regression analysis was conducted to obtain the fitting parameter *k_f_* = −0.156. The fitting result is shown in Figure 12.

(5) $$\begin{array}{ccc} \beta_{s}=0.545\left(\frac{l_{2.4}}{l_{g}}\right)+0.455 & & \left[0,1\right] \end{array}$$

where *l_g_* (mm) is the spacing of transverse steel strand. *l*_2.4_ is the reference spacing, which is 20 mm in this article.

Due to manufacturing errors, the mechanical properties of each specimen are different, and the test error cannot be completely eliminated. Future research can further validate the effectiveness of this prediction model.

## 6. Conclusions

A total of 39 specimens were fabricated and tested to investigate the lap-spliced performance, and were grouped into 13 sets. The effects of the transverse steel strand spacing, steel strand diameter, and lap length on the lap-spliced performance were analyzed based on experimental observations and results. The following main conclusions were drawn:

(1) Transverse steel strands in lap-spliced specimens can effectively limit the longitudinal steel strand from slipping. Within a certain range, smaller transverse steel strand spacing resulted in a smaller slip amount at the peak load;
(2) Lap strength was found to be negatively correlated with the diameter of steel strands, with larger diameters resulting in smaller lap strength when other factors were kept constant;
(3) A calculation formula for the lap strength of HSSSW in ECCs was proposed based on the test results and the calculation formula for the tensile strength of the interface between HSSSW and ECCs.

## Figures and Tables

**Table 1:** Mix proportion of ECCs.

Fly Ash	Silica Powder	Cement	Silica Sand	PVA Fiber	Water	Superplasticizer
2.5	0.093	1	0.4	0.02	1.15	0.058

**Table 2:** Geometric parameters and measured mechanical performance of HSSSW.

Diameter/mm	Measured Area/mm^2^	Young’s Modulus/GPa	Ultimate Tensile Strength/MPa	Mean Ultimate Tensile Strain/%	Ultimate Load/kN
4.5	9.62	108	1687.4	3.78	16.23
3.2	4.94	97	1589.2	4.08	7.87
2.4	2.82	130	1568.3	3.07	4.42

**Table 3:** The parameters of specimens.

Group		Specimen	Diameter of HSSSW (mm)	Splice Length (mm)	The Spacing of Transverse HSSSW (mm)	Size (mm^3^)
A	A-1	A-1-451500	4.5	15d	0	150 × 150 × 50
	A-2	A-2-451520	4.5	15d	20	
	A-3	A-3-451530	4.5	15d	30	
	A-4	A-4-451540	4.5	15d	40	
B	B-1	B-1-452030	4.5	20d	30	150 × 150 × 50
	B-2	B-2-452830	4.5	28d	30	150 × 200 × 50
	B-3	B-3-453230	4.5	32d	30	
C	C-1	C-1-322030	3.2	20d	30	150 × 150 × 37
	C-2	C-2-322230	3.2	22d	30	
	C-3	C-3-322530	3.2	25d	30	
D	D-1	D-1-242030	2.4	20d	30	150 × 100 × 27
	D-2	D-2-242230	2.4	22d	30	
	D-3	D-3-242530	2.4	25d	30	

**Table 4:** Experimental results.

Group		Specimen	Size (mm^3^)	Test Result				
				Failure Mode	*T_a_* (kN)	*τ_a_* (MPa)	*l_LP_* (mm)	*φ* (%)
A	A-1	A-1-451500-1	150 × 150 × 50	Pull-out	10.25	10.75	2.93	63.1
		A-1-451500-2		Pull-out	10.32	10.82	3.05	63.6
		A-1-451500-3		Pull-out	10.04	10.53	2.98	61.9
	A-2	A-2-451520-1	150 × 150 × 50	Pull-out	10.14	10.63	1.65	62.5
		A-2-451520-2		Pull-out	10.17	10.66	1.62	62.7
		A-2-451520-3		Pull-out	13.05	13.68	1.45	80.4
	A-3	A-3-451530-1	150 × 150 × 50	Pull-out	10.22	10.72	2.03	63
		A-3-451530-2		Pull-out	10.54	11.05	2.03	64.9
		A-3-451530-3		Pull-out	10.23	10.73	2.02	63
	A-4	A-4-451540-1	150 × 150 × 50	Pull-out	9.99	10.47	2.41	61.5
		A-4-451540-2		Pull-out	10.25	10.75	2.53	63.1
		A-4-451540-3		Pull-out	10.42	10.93	2.5	64.2
B	B-1	B-1-452030-1	150 × 150 × 50	Pull-out	11.7	9.2	2.23	72.1
		B-1-452030-2		Pull-out	11.7	9.2	2.18	72.1
		B-1-452030-3		Pull-out	11.38	8.95	2.22	70.1
	B-2	B-2-452830-1	150 × 200 × 50	Rupture	14.4	8.09	2.62	88.7
		B-2-452830-2		Pull-out	14.21	7.98	2.52	87.5
		B-2-452830-3		Pull-out	14.19	7.97	2.63	87.4
	B-3	B-3-453230-1	150 × 200 × 50	Rupture	14.35	7.05	2.79	88.4
		B-3-453230-2		Rupture	14.5	7.13	2.73	89.3
		B-3-453230-3		Rupture	14.29	7.02	2.78	88
C	C-1	C-1-322030-1	150 × 150 × 37	Pull-out	6.41	9.97	2.3	81.7
		C-1-322030-2		Pull-out	6.29	9.78	2.35	80.1
		C-1-322030-3		Pull-out	6.36	9.89	2.34	81
	C-2	C-2-322230-1	150 × 150 × 37	Pull-out	7	9.9	2.47	89.2
		C-2-322230-2		Pull-out	7.08	10.01	2.56	90.2
		C-2-322230-3		Pull-out	6.98	9.87	2.49	88.9
	C-3	C-3-322530-1	150 × 150 × 37	Rupture	7.71	9.59	2.64	98.2
		C-3-322530-2		Rupture	7.78	9.68	2.63	99.1
		C-3-322530-3		Rupture	7.79	9.69	2.65	99.2
D	D-1	D-1-242030-1	150 × 100 × 27	Pull-out	3.84	10.62	2.42	86.8
		D-1-242030-2		Pull-out	3.83	10.59	2.38	86.6
		D-1-242030-3		Pull-out	3.85	10.64	2.35	87
	D-2	D-2-242230-1	150 × 100 × 27	Pull-out	4.02	10.1	2.57	90.9
		D-2-242230-2		Pull-out	4.07	10.23	2.53	92
		D-2-242230-3		Pull-out	3.99	10.03	2.52	90.2
	D-3	D-3-242530-1	150 × 100 × 27	Pull-out	4.27	9.44	2.72	96.6
		D-3-242530-2		Pull-out	4.43	9.8	2.78	98.4
		D-3-242530-3		Pull-out	4.29	9.49	2.63	97

Note: The naming method of specimen, for example, the specimen “A-1-451500-1” represents the first specimen in group “A-1”, where the diameter of steel strand is 4.5 mm, the lap length is 15d, and the spacing of transverse steel strand is 0 mm. *T_a_* (kN) is the peak load, *τ_a_* (MPa) is the average bond stress calculated by formula (3), and *l_LP_* (mm) is the slip amount calculated by formula (2). *φ* (%) is the strength conversion rate for HSSSWM.

## Data Availability

The data presented in this study are available on request from the corresponding author.

## References

[1] Ahmed S.F.U., Mihashi H. (2007). A review on durability properties of strain hardening fibre reinforced cementitious composites (SHFRCC). Cem. Concr. Compos..

[2] Al-Quraishi H., Al-Farttoosi M., AbdulKhudhur R. (2019). Tension Lap Splice Length of Reinforcing Bars Embedded in Reactive Powder Concrete (RPC). Structures.

[3] Al-Salloum Y.A., Siddiqui N.A., Elsanadedy H.M., Abadel A.A., Aqel M.A. (2011). Textile-Reinforced Mortar versus FRP as Strengthening Material for Seismically Deficient RC Beam-Column Joints. J. Compos. Constr..

[4] Li V.C., Leung C.K.Y. (1992). Steady-state and multiple cracking of short random fiber composites. J. Eng. Mech.-ASCE.

[5] Chen J.X., Fang Z., Chen X., Jiang R.N. (2022). Experimental study on lap behavior of CFRP indented bars in UHPC. Constr. Build. Mater..

[6] Choi Y.C., Cho K.H., Bae B.I., Choi H.K. (2014). Experimental study on the performance of tensile lap-spliced GFRP rebars in concrete beam. Mag. Concr. Res..

[7] Dong Z., Deng M., Zhang Y., Ma P. (2021). Strengthening of unreinforced masonry walls against out-of-plane loads using carbon textile reinforced mortar optimized by short PVA fibers. Eng. Struct..

[8] Fang Y., Jinlong P., Zhun X., Leung C.K.Y. (2013). A comparison of engineered cementitious composites versus normal concrete in beam-column joints under reversed cyclic loading. Mater. Struct..

[9] Halvaei M., Jamshidi M., Latifi M., Ejtemaei M. (2020). Effects of volume fraction and length of carbon short fibers on flexural properties of carbon textile reinforced engineered cementitious composites (ECCs); an experimental and computational study. Constr. Build. Mater..

[10] Hung C.C., Su Y.F. (2016). Medium-term self-healing evaluation of Engineered Cementitious Composites with varying amounts of fly ash and exposure durations. Constr. Build. Mater..

[11] Kondraivendhan B., Pradhan B. (2009). Effect of ferrocement confinement on behavior of concrete. Constr. Build. Mater..

[12] Li K., Liu W.K., Zhang K., Wang X.L., Zhu J.T., Sheikh S. (2021). Bond behavior of stainless steel wire ropes embedded in engineered cementitious composites. Constr. Build. Mater..

[13] Xu G., Wang Q., Wei J., Liu D. (2011). Experimental study of behavior of lap splice of corrosive reinforcing bars in concrete. Eng. J. Wuhan Univ..

[14] Ma F.D., Deng M.K., Fan H.K., Yang Y., Sun H.Z. (2020). Study on the lap-splice behavior of post-yield deformed steel bars in ultra high performance concrete. Constr. Build. Mater..

[15] Tighiouart B., Benmokrane B., Mukhopadhyaya P. (1999). Bond strength of glass FRP rebar splices in beams under static loading. Constr. Build. Mater..

[16] Zemour N., Asadian A., Ahmed E.A., Benmokrane B., Khayat K.H. (2019). Experimental Study on Splice Strength of Glass Fiber-Reinforced Polymer Reinforcing Bars in Normal and Self-Consolidating Concrete. ACI Mater. J..

[17] Wu C., Hwang H.J., Ma G. (2022). Effect of stirrups on the bond behavior of lap spliced GFRP bars in concrete beams. Eng. Struct..

[18] Tabatabaei A., Eslami A., Mohamed H.M., Benmokrane B. (2018). Strength of compression lap-spliced GFRP bars in concrete columns with different splice lengths. Constr. Build. Mater..

[19] Wu C., Li V.C. (2017). CFRP-ECC hybrid for strengthening of the concrete structures. Compos. Struct..

[20] Zhu J.T., Zhang K., Wang X.L., Li K., Zou X.Y., Feng H. (2022). Bond-Slip Performance between High-Strength Steel Wire Rope Meshes and Engineered Cementitious Composites. J. Mater. Civ. Eng..

[21] Zhang K., Yuan Q., Huang T., Zuo S., Yao H. (2023). Utilization of novel stranded steel fiber to enhance fiber–matrix interface of cementitious composites. Constr. Build. Mater..

[22] Wang L.C., Yin S.P., Hua Y.T. (2021). Flexural behavior of BFRP reinforced seawater sea-sand concrete beams with textile reinforced ECC tension zone cover. Constr. Build. Mater..

[23] Wang Y., Hou M., Yu J., Xu S., Yu K., Zhang Z. (2018). Experimental Study on Mechanical Properties of Ultra-High Ductile Cementitious Composites. Mater. Rev..

[24] Zhang H.Y., Liu H.Y., Kodur V., Li M.Y., Zhou Y. (2022). Flexural behavior of concrete slabs strengthened with textile reinforced geopolymer mortar. Compos. Struct..

[25] Zhang Q., Wei Z.Y., Gu X.L., Yang Q.C., Li S.Y., Zhao Y.S. (2022). Confinement behavior and stress-strain response of square concrete columns strengthened with carbon textile reinforced concrete (CTRC) composites. Eng. Struct..

[26] Zhou J.F., Stuempel M., Kang C.J., Marx S. (2022). Lap-spliced connections of steel and FRP bars in reinforced flexure concrete structures. Eng. Struct..

[27] Meng D., Lee C.K., Zhang Y.X. (2017). Flexural and shear behaviours of plain and reinforced polyvinyl alcohol-engineered cementitious composite beams. Eng. Struct..

[28] Meng D., Zhang Y.X., Lee C.K. (2019). Flexural fatigue behaviour of steel reinforced PVA-ECC beams. Constr. Build. Mater..

[29] Metelli G., Marchina E., Plizzari G.A. (2017). Experimental study on staggered lapped bars in fiber reinforced concrete beams. Compos. Struct..

[30] Mousavi S.S., Dehestani M., Mousavi S.M. (2016). Bond strength and development length of glass fiber-reinforced polymer bar in unconfined self-consolidating concrete. J. Reinf. Plast. Compos..

[31] Najafgholipour M.A., Dehghan S.M., Khani M., Heidari A. (2018). The performance of lap splices in RC beams under inelastic reversed cyclic loading. Structures.

[32] Nie J., Tao W., Zhang T. (2007). Experimental study on the flexural behavior of RC beams strengthened with prestressed stainless steel wire mesh and permeability polymer mortar. China Civ. Eng. J..

[33] Parra-Montesinos G., Wight J.K. (2000). Seismic response of exterior RC column-to-steel beam connections. J. Struct. Eng..

[34] Si Z.H., Liu F., Pan J.W., Dong H. (2022). Research on Impact Resistance of Reinforced Concrete Beams Strengthened with Carbon Fiber Reinforced Polymer Grid and Engineered Cementitious Composites. Polymers.

[35] Wang X.L., Yang G.H., Qian W.W., Li K., Zhu J.T. (2021). Tensile Behavior of High-Strength Stainless Steel Wire Rope (HSSSWR)-Reinforced ECC. Int. J. Concr. Struct. Mater..

[36] Li K., Zhao D.P., Fan J.J., Zhu J.T. (2022). Local Bond Stress-Slip Model of High-Strength Stainless Steel Wire Ropes in ECC. KSCE J. Civ. Eng..

[37] Xu S., Li Q., Li H. (2007). An experimental study on the flexural properties of carbon textile reinforced ECC. China Civ. Eng. J..

[38] Yang Y.Z., Lepech M.D., Yang E.H., Li V.C. (2009). Autogenous healing of engineered cementitious composites under wet-dry cycles. Cem. Concr. Res..

[39] Yu K.Q., Li L.Z., Yu J.T., Wang Y.C., Ye J.H., Xu Q.F. (2018). Direct tensile properties of engineered cementitious composites: A review. Constr. Build. Mater..

[40] Yuan F., Chen M.C., Pan J.L. (2020). Flexural strengthening of reinforced concrete beams with high-strength steel wire and engineered cementitious composites. Constr. Build. Mater..

[41] Zhu J., Liu Y., Li Z., Zou X., Li K., Fan J. (2023). Bond behavior between high-strength steel wire meshes and ECC: Experimental study and analytical modelling. Eng. Struct..

[42] Zhu J., Liu Y., Wang J., Li K. (2022). Experimental on bond properties of grooved interface between high-strength steel wire mesh reinforced ECC and concrete. J. Compos. Mater..

